# Can learning presence be the fourth community of inquiry presence? Examining the extended community of inquiry framework in blended learning using confirmatory factor analysis

**DOI:** 10.1007/s10639-022-11480-z

**Published:** 2022-11-30

**Authors:** Ghada ElSayad

**Affiliations:** Business Information Systems, College of Management and Technology, Arab Academy for Science, Technology and Maritime Transport (AAST), Heliopolis, Cairo Egypt

**Keywords:** Blended learning, Learning presence, Self-regulation, Community of Inquiry

## Abstract

Researchers continue to extend the community of inquiry (COI) framework, highlighting its utility in online and blended learning environments for providing a successful learning experience. Recent studies have added the learning presence dimension to the classic COI framework which contains teaching, social, and cognitive presences, to represent online students’ traits of self-regulation. However, there is a need to examine whether this additional presence structurally represents relationships with other COI presences. Attempting to fill this gap, this study examines the statistical structure of the extended COI framework (integrating the classic COI presences with the additional learning presence) as well as the structural path between the four presences, using confirmatory factor analysis (CFA). Data were collected from 205 undergraduate students who were enrolled in blended courses during the COVID-19 pandemic. Study findings revealed that learning presence has strong correlations with the classic COI counterparts, especially cognitive presence. Furthermore, learning presence has significant positive relationships with cognitive presence and social presence. Overall, the validity and reliability of the extended COI framework (which integrates the classic COI presences with the additional learning presence) had been proven in this study. This study contributes to the literature by providing a comprehensive framework of the extended COI framework, proving their multi-dimensionality and inter-relationality.

## Introduction

The COI framework is considered one of the widely validated and accepted frameworks which represents the learning community in online and blended learning environments (Cleveland-Innes, 2019; Stenbom, 2018; Wertz, 2022). It relies on socio-constructivism and reflective thinking and practical inquiry perspectives and, thus, it is considered a robust framework for learning design and inquiry (Cherney et al., 2017; Tolu, 2013). It was conducted to help educators in understanding how to build a successful learning community which is mediated by computer and communication technologies (Garrison, 2007; Garrison et al., 1999). The classic COI framework comprises three overlapping presences: teaching presence (TP), social presence (SP), and cognitive presence (CP) (Garrison et al., 1999). TP describes interactions between the instructor with students and course content (Anderson et al., 2001). SP describes the generation of a learning community that encourages students to express themselves to fellow students, ask questions, negotiate their perspectives, and express their ideas in order to foster their belonging (Garrison, 2016; Garrison et al., 1999). CP describes the process of constructing students’ meaning and understanding as well as knowledge acquisition and application (Garrison & Vaughan, 2008; Garrison et al., 1999).

COI is considered a remarkable area of interest which suggests that online and blended learning participants must focus on creating social and knowledge processes through their interactions and negotiations which are occurred in the face-to-face classrooms (Garrison et al., 1999; Wertz, 2022). Studies have demonstrated that shifting the face-to-face learning to computer-based learning has an impact on students’ grades, engagement, well-being, and withdrawal rates (Asarta & Schmidt, 2017; Murphy & Stewart, 2017; Zhao et al., 2022). Thus, it is important to understand the methods that help in supporting higher education students to successfully cope with this learning experience (Gnaur et al., 2020).

COI presences have been proven to have a significant impact on students’ satisfaction, learning perception, and course grades in online and blended learning environments (Lee et al., 2021; Maddrell et al., 2017; Shea et al., 2012). Researchers argued that the COI framework does not include enough attention to the roles of students’ learning experience and involvement in such learning environments (Shea & Bidjerano, 2010; Shea et al., 2012). Students encounter more autonomy in computer-mediated learning environments which required them to devote efforts to apply self-regulation skills (Pool et al., 2017). They expand efforts in their discussions through managing their time, dividing up the given tasks, and setting goals to successfully complete these tasks (Shea & Bidjerano, 2010). Self-regulation is a self-directive process by which students transfer their possessed mental skills into their academic skills (Zimmerman, 2002, p. 65). It has an important role in explaining students’ learning experience as it reflects students’ systematic effort to direct thoughts, emotions, and behaviours for managing their learning process and achieving desired outcomes (Cho & Heron, 2015; Zimmerman, 2002; Zimmerman & Schunk, 2011). Drawn from this perspective, literature introduced learning presence (LP) as a fourth presence of the COI framework to reflect online students’ self-regulation process (Shea & Bidjerano, 2010, 2012; Shea et al., 2012; Wertz, 2014, 2022). Furthermore, previous studies emphasised the significant role of integrating learning presence with the classic COI presences in developing a successful blended learning environment (Shea & Bidjerano, 2010, 2012; Traver et al., 2014).

Many studies have introduced LP to reflect motivational and behavioural traits of self-regulation and co-regulation, suggesting that students are intending to accomplish desired goals, which is not considered in the classic COI framework (Hayes et al., 2015; Shea & Bidjerano, 2010; Shea et al., 2012, 2014). However, as the self-regulation concept reflects the extent to which students behaviourally, motivationally, and metacognitively act as active participants in their learning process (Zimmerman, 2008), the intellectual development which is driven from the self-regulation process should not be neglected. Learning can be constructed by the individual and the social environment (Wertz, 2022). Self-regulation was found to have a significant influence on online students’ intellectual learning (Eom & Ashill, 2018). According to this, Wertz (2014, 2022) argued that LP comprises motivational, behavioural, and development dimensions which are integrated to reflect online students’ self-regulation process.

Although researchers attempt to extend the COI framework and gain a deeper understanding of its factors (presences), there is a need to prove that it has a fourth presence (Wertz, 2022). Besides, the structural model that represents the relationships between this additional presence with the classic COI presences should be statistically proven. More specifically, there are many arguments regarding whether the LP dimension has a structural relationship with other COI dimensions, considering their multi-dimensionality (Kozan & Caskurlu, 2018; Wertz, 2022). Hence, there is a need to examine the multi-dimensionality of the four COI dimensions in one model and their relationships together in a comprehensive study (Wertz, 2022). Thus, using the confirmatory factor analysis (CFA), this study pursues to examine the validity and reliability, the dimensionality structure, the whole structure, and the structural path between the four presences of the extended COI framework (TP, SP, CP, and LP), concerning LP’s motivational, behavioural, and development dimensions.

## The classic COI framework

The classic COI framework comprises three overlapping presences: TP, SP, and CP (Garrison, 2007; Garrison et al., 1999). TP illustrates to which extent course instructors design, facilitate, and direct students’ cognitive and social processes to attain meaningful learning outcomes (Anderson et al., 2001). It comprises three sub-dimensions: instructional design and organization, discourse facilitation, and direct instructions (Garrison et al., 1999). The design and organization sub-dimension describes instructors’ design of course contents, the utilized teaching approaches, and the applied adjustments (if needed). The discourse facilitation sub-dimension illustrates instructors’ roles in facilitating students’ understanding of course topics, encouraging their thinking, supporting their participation in a dialogue for sharing knowledge, keeping them on learning tasks, exploring new concepts, and developing the community sense between them. The direct instruction sub-dimension describes instructors’ handling of specific issues such as illustrating the difficult points, guiding students’ misconceptions, and providing students with timely feedback that clarifies their strengths and weaknesses (Garrison, 2016).

SP illustrates students’ ability to express themselves socially and emotionally to fellow students through the medium communication means (Garrison et al., 1999). It comprises three sub-dimensions: affective expression, open communication, and group cohesion. The affective expression describes students’ seeking to know course participants, open communication refers to the trust and comfort climate which is required to allow students to express their agreement and disagreement points, and group cohesion help in sustaining the community sense by which students collaborate and share their understanding to attain desired learning outcomes (Garrison, 2016).

CP is considered the core of COI which reflects students’ ability to reflect, construct, and build meaning from their participation in reflective practices of inquiry: triggering event, exploration, integration, and resolution (Garrison & Arbaugh, 2007; Garrison et al., 1999). The triggering event practice reflects students’ recognition of the problem and the sense of interest, motivation, and curiosity generated by a given task. The exploration practice refers to gathering information and ideas about the problem from the different resources and through students’ discussions together. The integration practice describes students’ reflection and construction of meaningful solutions and explanations, while the resolution process practice refers to evaluating the effectiveness of the problem-solving process. In other words, CP can be summarized by four stages: 1) student’s comprehension of a problem, 2) student’s discussion with fellow students about this problem, 3) student’s construction of meanings from the information acquired from this discussion, and 4) solving this problem through consensus building. These stages are considered essential for obtaining deep and meaningful learning (Garrison et al., 1999).

The validity of the classic COI framework has been proven by researchers in asynchronous and synchronous learning environments (Garrison & Arbaugh, 2007; Swan et al., 2008). In addition, strong correlations between COI presences together have been indicated in both online and blended learning studies (Heilporn & Lakhal, 2020; Maddrell et al., 2017; Zhang, 2020).

## The extended COI framework

The extended COI framework includes LP with the three classic COI counterparts (Shea & Bidjerano, 2010, 2012; Shea et al., 2012; Wertz, 2014, 2022). The LP dimension was firstly introduced by Shea and Bidjerano (2010) to describe students’ self-regulation process in online and blended learning environments. Authors considered self-efficacy and effort regulation as sub-dimensions of LP which are driven by students’ engagement with the course and their collaborative attempts to understand the provided instructions by their instructors. The findings revealed that self-efficacy and effort regulation have positive and significant correlations with COI presences in online and blended learning environments. Likewise, Traver et al. (2014) used a pre/post-test COI survey to investigate students’ perceptions of COI presences in courses conducted in the blended learning approach and found that LP is positively and significantly correlated with the other COI presences. Furthermore, Shea and Bidjerano (2012) and Shea et al. (2012) used the self-regulation sub-dimensions (namely, environmental structuring, goal Setting, time management, help-seeking, task strategies, self-evaluation, forethought/planning, monitoring, and strategy use) to examine the correlation between LP and COI presences in online and blended learning environments, and indicated positive and significant correlations.

Wertz (2014, 2022) argued that LP comprises three sub-dimensions: motivation, behaviour, and development. Motivation refers to students’ activation and persistence in a selected behaviour as well as their belief in their skills to persist or try to develop strategies for a given situation (Bandura, 1977). The behaviour sub-dimension reflects student usage of self-regulation behaviours, while the development sub-dimension describes students’ intellectual development process which is an explicit indicator of their self-regulation abilities (Wertz, 2014, 2022). The findings showed positive and significant correlation and path coefficient estimates between LP and COI presences.

## Study method

### Confirmatory factor analysis

Confirmatory factor analysis (CFA) is used to statistically examine the hypothesized structural model of study constructs based on the extended COI concept. Its procedures were performed to evaluate the lower-order constructs (LOCs), confirming their measurement models, as well as the higher-order constructs (HOCs) to confirm the structure of the extended COI framework (all four presences are included in one model). HOCs represent the main COI presences, while LOCs comprise their sub-dimensions. CFA procedures were deployed using the IBM SPSS AMOS software (version 22). The analysis procedures comprised multivariate normality of study data, factors loadings, correlation estimates, model fit indices, and modification indices (MIs). Initial models were firstly evaluated by the indices of model fit and MIs, and this was repeated till meeting acceptable levels of model fit.

### Instruments

The survey of this study comprised two sections, one section included questions regarding participant demographics (age, gender, and courses enrolled in) and the other section included 54 COI items to investigate participants’ perceptions of teaching, social, learning, and cognitive presences in blended learning. Teaching, social, and cognitive presences scales were pre-developed by Arbaugh et al, (2008), Swan et al., (2008), and Shea and Bidjerano (2010). The TP scale comprised 13 items regarding students’ perceptions of the instructor’s role in designing and organizing the course, facilitating their learning, and guiding them by providing helpful instructions. Therefore, the TP scale had three subscales which are design and organization (DO), discourse facilitation (DF), and direct instructions (DI), with four items, six, and three items, respectively. The SP scale included nine items reflecting the extent to which students have a sense of community and that they are connected to their fellow students. It comprised three subscales which are affective expression (AE), open communication (OC), and group cohesion (GC), with three items in each subscale. The CP scale included 12 items to capture students’ perceptions of their ability to construct their learning experience by applying inquiry activities within a sustainable community. It included four subscales (with three items for each), Triggering event (TE), exploration (EX), integration (IN), and Resolution (RE).

The LP scale was developed by Wertz (2014, 2022), with 20 items capturing students’ perceptions of their motivational, behavioural, and development traits which can be derived from their self-regulation activities. It involved the motivation (MO), behaviour (BH), and development (DE) subscales, with five, eight, and seven items, respectively. All COI items were ranked on a five-point Likert scale ranging from 1 = strongly disagree to 5 = strongly agree. To avoid obtaining missing data, the required-answer option was applied to all questions of the survey.

### Data collection, study context, and participants

The survey was sent to students who were enrolled in the college of management and technology, at a higher educational institution in Egypt, via their email. Students experienced courses with the blended learning approach during the first semester of 2021/2022, during the COVID-19 pandemic. Each course was divided into several groups of students, allowing to apply social distance precautions. Furthermore, students had the opportunity to attend their courses two times per week in face-to-face and fully online methods. Courses materials were available on the Moodle platform including electronic books, lessons’ presentations, instructors’ recorded videos, and individual and group weekly assignments. Besides, synchronous lectures (for two hours) were conducted once a week for each course to allow students’ and instructors’ discussions.

The data collection process yielded with receiving 205 valid responses. All students were undergraduates and experienced online learning since the breakout of the pandemic. 44.9% of students were aged between 16 and 20 years old, 53.7% were between 21 and 24 years old, while 1.5% were above 24 years old. Furthermore, students were of both genders: males and females, with 65.4% and 34.6%, respectively. Lastly, students were enrolled in different courses: 46.3% in social media, 22.4% in web design fundamentals, 18.5% in information retrieval and search engines, 7.8% in introduction of information systems (1), and 4.9% in e-commerce technologies. This indicates that most students were drawn from the business information systems major.

## Results

Before conducting CFA procedures, the multivariate normality of study constructs was evaluated. Results demonstrated that Mardia’s normalized values ranged from 63.6 to 225.9 (exceeding the value of 5) which were considered multivariate non-normal (Bentler & Wu, 2005). Dealing with this, bootstrapping of 95% procedures were applied to evaluate parameter estimates with their confidence intervals (CI). Furthermore, the adequacy of sampling and data were inspected by conducting exploratory factor analysis without extracting any number of factors. The correlation matrix yielded with large values exceeded the value of 0.30, indicating the suitability of data for factor analysis. Besides, the obtained Kaiser–Meyer–Olkin (KMO) and Bartlett's test of sphericity (BTS) values were 0.938 and ${X}^{2}\left(1431\right)=9685.706$, $p=0.000$, respectively, proofing the sampling adequacy for factor analysis and the good fit of study instruments (Field, 2009; Tabachnick et al., 2019).

### Lower-order measurement models of COI presences

**Fig. 1:**
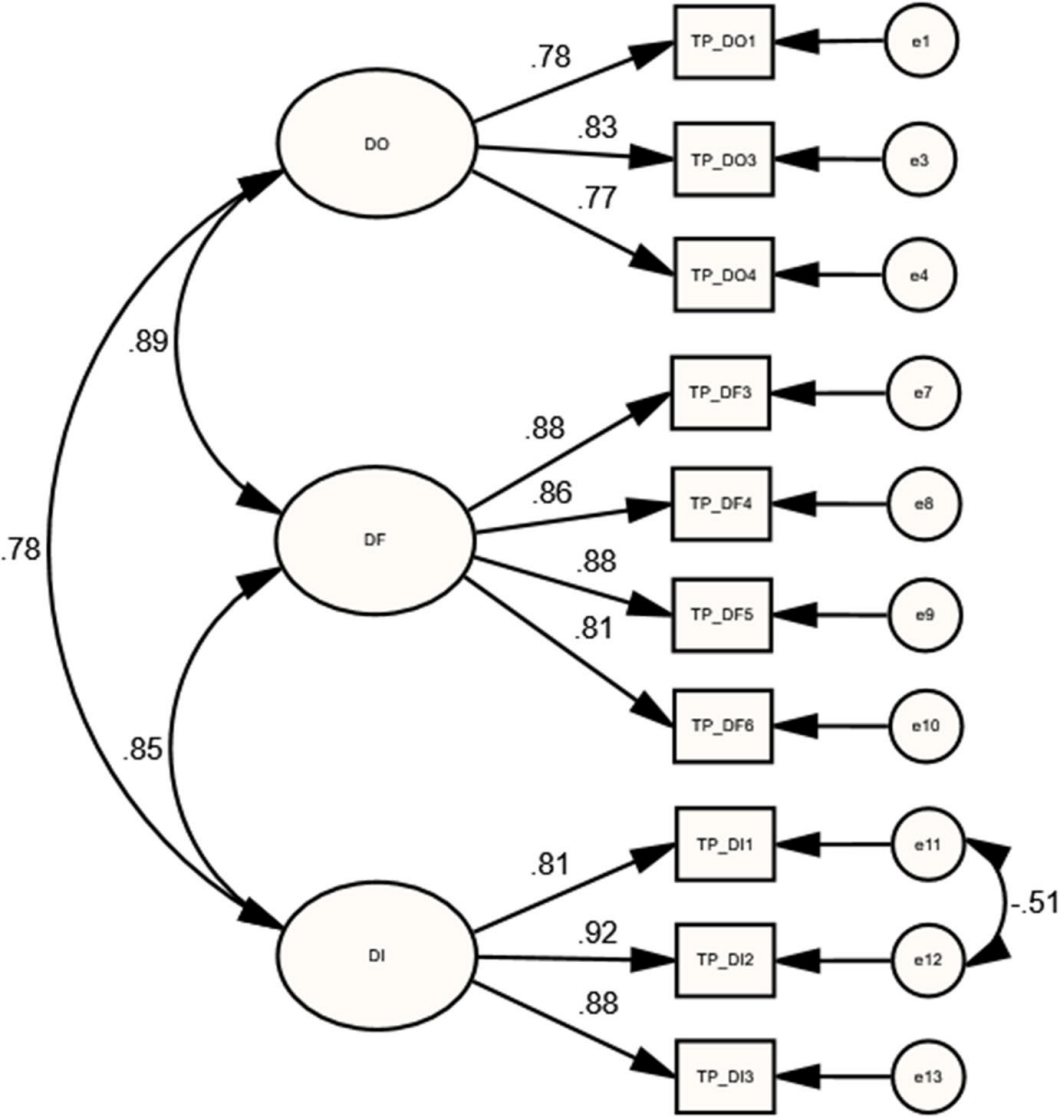
Standardized estimates of the final TP model

As presented in Table 1, the initial results of lower-order COI presences’ models showed mediocre and poor model fits. Accordingly, the MIs of each lower-order model were reviewed and further specifications were applied. For the lower-order TP model, the MIs evaluation yielded correlating two residuals (errors) of TP_DI1 and TP_DI2 indicators which had a strong correlation, as suggested by Byrne (2001). Moreover, three indicators (TP_DO2, TP_DF1, and TP_DF2) were dropped because of their high residual values and they were cross-loaded on other items and constructs. As a result, the final model (with 10 indicators) yielded an acceptable model fit [${X}^{2}(31)= 66.97$; $P=0.000$; $CMIN/DF=2.16$; $GFI=0.94$; $IFI=0.98$; $CFI=0.98$; $TLI=0.97$; $RMSEA=0.075$]. Results also showed strong correlations between DO and DF ($r=0.886$, $p<0.001$), DF and DI ($r=0.848$, $p<0.05$), and DO and DI ($r=0.782$, $p<0.05$), as shown in Table 2. In addition, the regression weight estimates showed significant factor loadings of sub-dimension’s indicators, as illustrated in Fig. 1, Table 3.

**Table 1:** Model fit results

Indices	Accepted criteria	TP		SP		LP		CP	
		Initial	Final	Initial	Final	Initial	Final	Initial	Final
X^2^	• > 0.05, indicating good fit. However, it is very sensitive to sample size (Bollen, 1989)	285.32	66.97	131.82	37.92	451.09	201.83	136.20	104.99
DF		62	31	24	16	167	109	48	38
P		0.00	0.00	0.00	0.00	0.00	0.00	0.00	0.00
CMIN/DF	• < 5 shows acceptable model fit, even if ${x}^{2}$ is significant ($p<0.05$)	4.60	2.16	5.49	2.37	2.70	1.85	2.84	2.76
GFI	• ≥ 0.90 (Timothy, 2015) • ≥ 0.95 (Carlson & Mulaik, 1993; Schreiber et al., 2006)	0.81	0.94	0.87	0.96	0.82	0.94	0.90	0.92
IFI		0.91	0.98	0.90	0.98	0.88	0.96	0.95	0.96
CFI		0.90	0.98	0.90	0.98	0.88	0.96	0.95	0.96
TLI	• ≥ 0.95 (Carlson & Mulaik, 1993; Schreiber et al., 2006)	0.88	0.97	0.85	0.96	0.86	0.95	0.93	0.94
RMSEA	• < 0.05, good fit (Browne & Cudeck, 1993) • < 0.06, acceptable (Brown, 2015; Browne & Cudeck, 1993; Hu & Bentler, 1999) • From 0.08 to 0.10, mediocre fit (MacCallum et al., 1996) • > 0.10, poor fit (Browne & Cudeck, 1993)	0.133	0.075	0.148	0.080	0.091	0.075	0.095	0.090

$${X}^{2}=\mathrm{Chi}-\mathrm{squared}$$; $$DF=Difference degree$$; $$GFI=Goodness of fit$$; $$IFI= Incremental Fit Index$$; $$CFI=comparative fit index$$; $$RMSEA=root mean square error of approximation$$

**Table 2:** Correlations of each presence’s LOCs

COI presences	Parameters	Estimates	Bootstrapping CI 95%		P
			Lower	Upper	
TP	DO $\leftrightarrow$ DF	0.886**	0.791	0.963	0.006
	DF $\leftrightarrow$ DI	0.848*	0.650	0.916	0.036
	DO $\leftrightarrow$ DI	0.782*	0.660	0.881	0.013
SP	OC $\leftrightarrow$ GC	0.952*	0.854	1.025	0.020
	AE $\leftrightarrow$ GC	0.775*	0.635	0.895	0.013
	AE $\leftrightarrow$ OC	0.833**	0.724	0.942	0.006
LP	BH $\leftrightarrow$ DE	0.852*	0.74	0.939	0.012
	MO $\leftrightarrow$ BH	0.917*	0.851	0.993	0.010
	MO $\leftrightarrow$ DE	0.754**	0.603	0.894	0.008
CP	IN $\leftrightarrow$ RE	0.899**	0.814	1.002	0.006
	EX $\leftrightarrow$ IN	0.950**	0.879	1.064	0.005
	TE $\leftrightarrow$ EX	0.975**	0.904	1.047	0.007
	EX $\leftrightarrow$ RE	0.951*	0.884	1.021	0.011
	TE $\leftrightarrow$ IN	0.879*	0.754	0.973	0.018
	TE $\leftrightarrow$ RE	0.916*	0.822	0.981	0.014

$$N=205$$; *$$p<0.05$$; **$$p<0.01$$

**Table 3:** Standardized Regression Weights of lower-order COI presences’ measurement models

COI presences	Parameters	Estimates	Bootstrapping CI 95%		P
			Lower	Upper	
TP	TP_DO1 $\leftarrow$ DO	0.776*	0.639	0.867	0.015
	TP_DO3 $\leftarrow$ DO	0.828*	0.731	0.882	0.028
	TP_DO4 $\leftarrow$ DO	0.766*	0.659	0.845	0.016
	TP_DF3 $\leftarrow$ DF	0.879*	0.815	0.908	0.034
	TP_DF4 $\leftarrow$ DF	0.858*	0.767	0.905	0.018
	TP_DF5 $\leftarrow$ DF	0.878*	0.805	0.918	0.023
	TP_DF6 $\leftarrow$ DF	0.814*	0.702	0.878	0.021
	TP_DI1 $\leftarrow$ DI	0.814**	0.706	0.894	0.009
	TP_DI2 $\leftarrow$ DI	0.919**	0.869	0.981	0.003
	TP_DI3 $\leftarrow$ DI	0.884*	0.820	0.931	0.019
SP	SP_AE1 $\leftarrow$ AE	0.871*	0.796	0.938	0.012
	SP_AE2 $\leftarrow$ AE	0.813*	0.739	0.887	0.012
	SP_OC1 $\leftarrow$ OC	0.648*	0.533	0.762	0.013
	SP_OC2 $\leftarrow$ OC	0.801**	0.725	0.898	0.007
	SP_OC3 $\leftarrow$ OC	0.872**	0.813	0.943	0.005
	SP_GC1 $\leftarrow$ GC	0.830**	0.715	0.917	0.007
	SP_GC2 $\leftarrow$ GC	0.807*	0.696	0.884	0.020
	SP_GC3 $\leftarrow$ GC	0.551*	0.374	0.728	0.013
LP	LP_MO1 $\leftarrow$ MO	0.673**	0.559	0.789	0.006
	LP_MO4 $\leftarrow$ MO	0.812*	0.733	0.878	0.012
	LP_MO5 $\leftarrow$ MO	0.841**	0.774	0.906	0.007
	LP_BH2 $\leftarrow$ BH	0.697**	0.561	0.823	0.004
	LP_BH3 $\leftarrow$ BH	0.758*	0.643	0.830	0.014
	LP_BH5 $\leftarrow$ BH	0.744**	0.647	0.826	0.007
	LP_BH7 $\leftarrow$ BH	0.732**	0.653	0.822	0.004
	LP_DE4 $\leftarrow$ DE	0.682*	0.558	0.784	0.010
	LP_DE5 $\leftarrow$ DE	0.825**	0.738	0.937	0.008
	LP_DE6 $\leftarrow$ DE	0.664*	0.490	0.780	0.012
CP	CP_TE1 $\leftarrow$ TE	0.759*	0.627	0.856	0.018
	CP_TE2 $\leftarrow$ TE	0.776**	0.698	0.867	0.007
	CP_TE3 $\leftarrow$ TE	0.813*	0.751	0.864	0.016
	CP_EX1 $\leftarrow$ EX	0.828*	0.692	0.888	0.018
	CP_EX2 $\leftarrow$ EX	0.785*	0.676	0.843	0.020
	CP_EX3 $\leftarrow$ EX	0.728**	0.641	0.800	0.009
	CP_IN1 $\leftarrow$ IN	0.827*	0.726	0.884	0.014
	CP_IN2 $\leftarrow$ IN	0.858*	0.779	0.914	0.025
	CP_RE1 $\leftarrow$ RE	0.848*	0.774	0.905	0.012
	CP_RE2 $\leftarrow$ RE	0.771*	0.681	0.842	0.010
	CP_RE3 $\leftarrow$ RE	0.793**	0.709	0.849	0.009

$$N=205$$; *$$p<0.05$$; **$$p<0.01$$

**Fig. 2:**
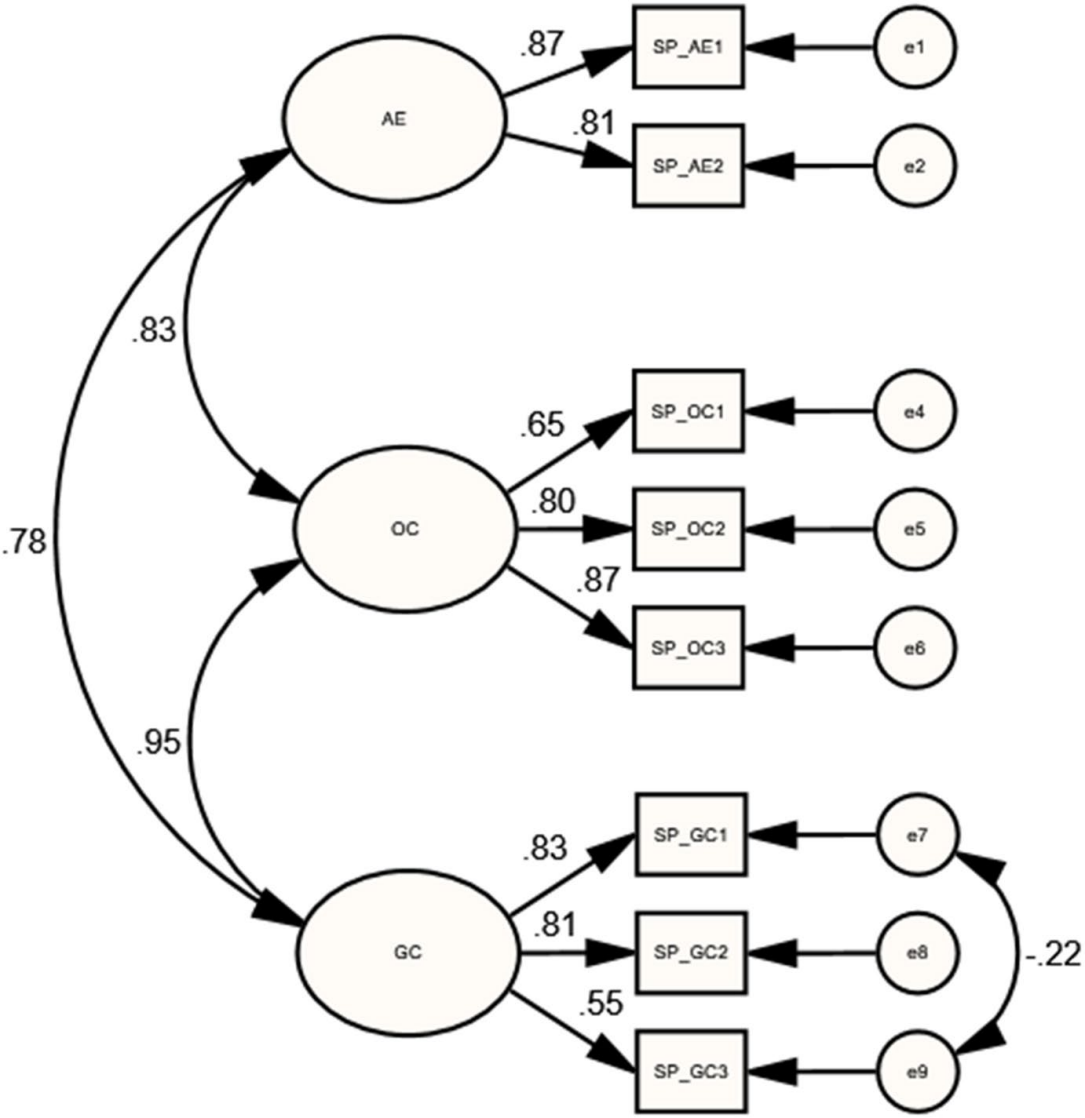
Standardized estimates of the final SP model

For SP lower-order model, the MIs showed that the residuals of two indicators (SP_GC1 and SP_GC3) had strong correlations. Furthermore, the SP_AE3 indicator was dropped because of its cross-loading on the other sub-dimensions. The final SP model, with 8 indicators, had obtained an acceptable model fit [${X}^{2}(16)=37.92$; $P=0.002$; $CMIN/DF=2.37$; $GFI=0.96$; $IFI=0.98$; $CFI=0.98$; $TLI=0.96$; $RMSEA=0.080$], as shown in Table 1. Furthermore, as illustrated in Table 2, results showed strong correlations between OC and GC ($r=0.952$, $p<0.05$), AE and GC ($r=0.775$, $p<0.05$), and even between AE and OC ($r=0.833$, $p<0.01$). Additionally significant factor loadings of each sub-dimension’s indicators were obtained, as illustrated in Fig. 2, Table 3.

**Fig. 3:**
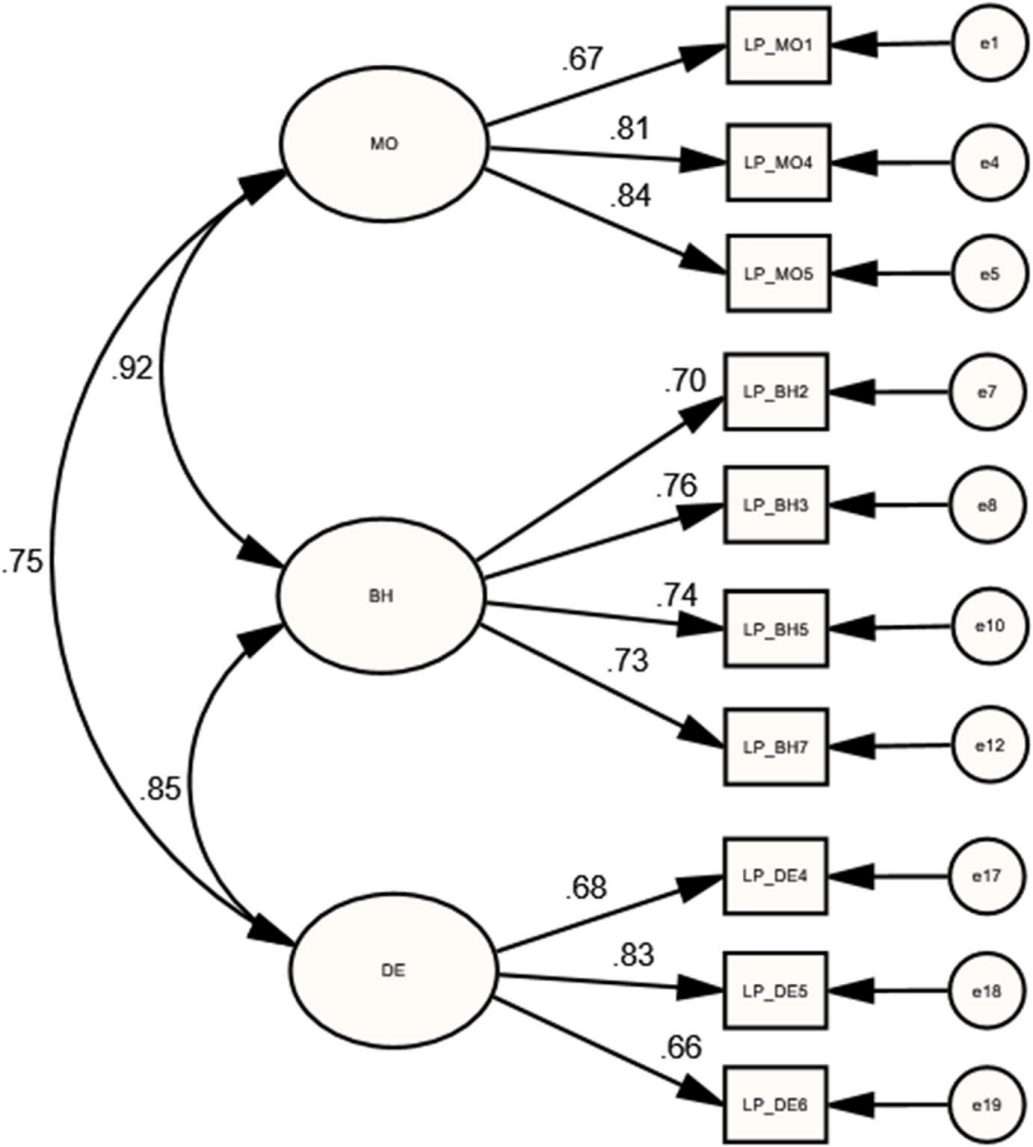
Standardized estimates of the final LP model

The MIs revision of the initial lower-order LP model resulted with dropping 10 indicators (LP_MO2, LP_MO5, LP_BH1, LP_BH4, LP_BH6, LP_BH8, LP_DE1, LP_DE2, LP_DDE3, and LP_DE7) indicators to meet a good model fit requirements [${X}^{2}(109)=201.83$; $P=0.000$; $CMIN/DF=1.85$; $GFI=0.94$; $IFI=0.96$; $CFI=0.96$; $TLI=0.95$; $RMSEA=0.075$], as presented in Table 1. As articulated by Wertz (2022), the current form of LP scale is recently developed and it is anticipated to eliminate several items which have weak performance till obtaining high-performing items in each subscale. As can be seen in Table 2, the final lower-order LP model shows that BH is strongly correlated with DE ($r=0.852$, $p<0.05$), MO with BH ($r=0.917$, $p at 0.01$), and MO with DE ($r=0.754$, $p<0.01$). Additionally, as presented in Fig. 3, Table 3, the final model comprised high and significant factor loadings.

**Fig. 4:**
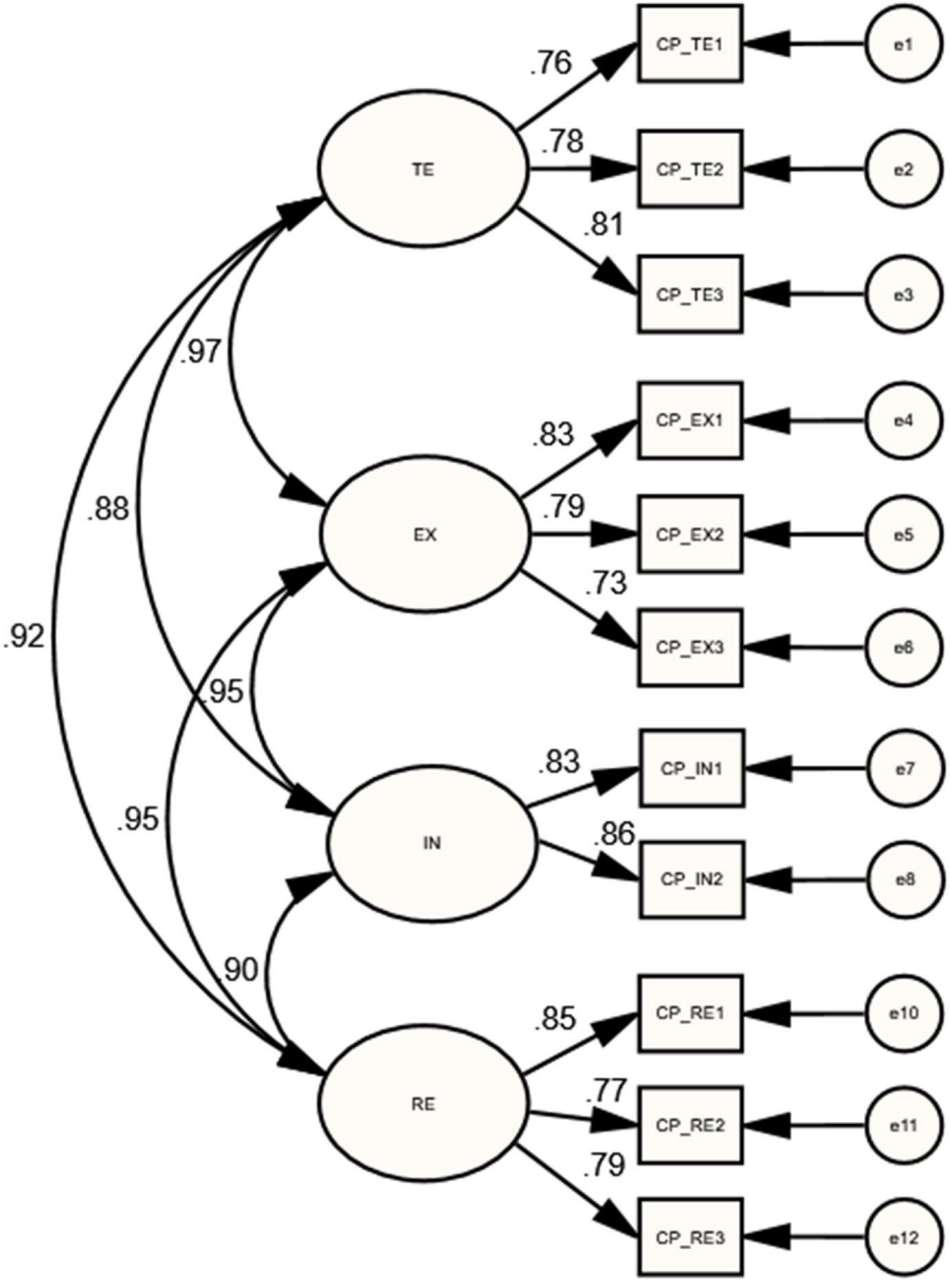
Standardized estimates of the final CP model

Lastly, for the lower-order CP model, the MIs yielded with dropping the CP_IN3 indicator because it cross-loaded on the RE construct. As presented in Table 1, the final model showed a mediocre model fit [${X}^{2}(38)=104.99$;$P=0.000$;$CMIN/DF=2.763$;$GFI=0.92$; $IFI=0.96$; $CFI=0.96$;$TLI=0.94$;$RMSEA=0.090$]. In addition, as shown in Table 2, results revealed that TE strongly correlated with EX ($r=0.975$, $p<$ 0.01), IN ($r=0.879$,$p<0.05$), and RE ($r=0.916$,$p<0.05$). IN strongly correlated with RE ($r=0.899$,$p<0.01$), while EX strongly correlated with IN ($r=0.950$,$p<0.01$) and RE ($r=0.951$,$p<0.05$). Moreover, as illustrated in Fig. 4, Table 3, indicators’ factors loading of each sub-dimension were high and significant.

### The higher-order measurement model of the extended COI framework

After confirming the measurement model of each lower-order model, the higher-order of COI framework was examined. Prior to that, to avoid the over-deletion of indicators in the higher-order analysis because of potential outliers’ presence, the Mahalanobis distance (D^2^) values were inspected, as recommended by Byrne (2001). Results revealed two outlier cases to be dropped, remaining 203 observations for the higher-order analysis. Additionally, Byrne's (2001) recommended procedures of the critical ratio of differences (CRDIFF) were applied to identify and constrain residual variance parameters of the higher-order model which could negatively influence the model fit.

The analysis of the higher-order COI framework comprised the four presences with their sub-dimensions. Results of the initial higher-order model showed poor model fit [${X}^{2}(690)=1420.16$;$P=0.000$;$CMIN/DF=2.058$;$IFI=0.89$; $CFI=0.89$;$TLI=0.88$;$RMSEA=0.072$], indicating the need for additional specifications to be applied. The MIs showed that SP_GC3 and TP_DF6 cross-loaded on other sub-dimensions, implying the need for dropping these indicators. Furthermore, strong correlations between the residuals of DF (res2) and DE (res9), DF (res2) and TE (res10), DI (res3) and IN (res12), and even between AE (res4) and IN (res12), ending up with correlating them. After applying these specifications, the final higher-order model yielded an acceptable model fit [${X}^{2}(613)=1129.72$;$P=0.000$;$CMIN/DF=1.843$;$IFI=0.91$; $CFI=0.91$;$TLI=0.91$;$RMSEA=0.065$].

**Fig. 5:**
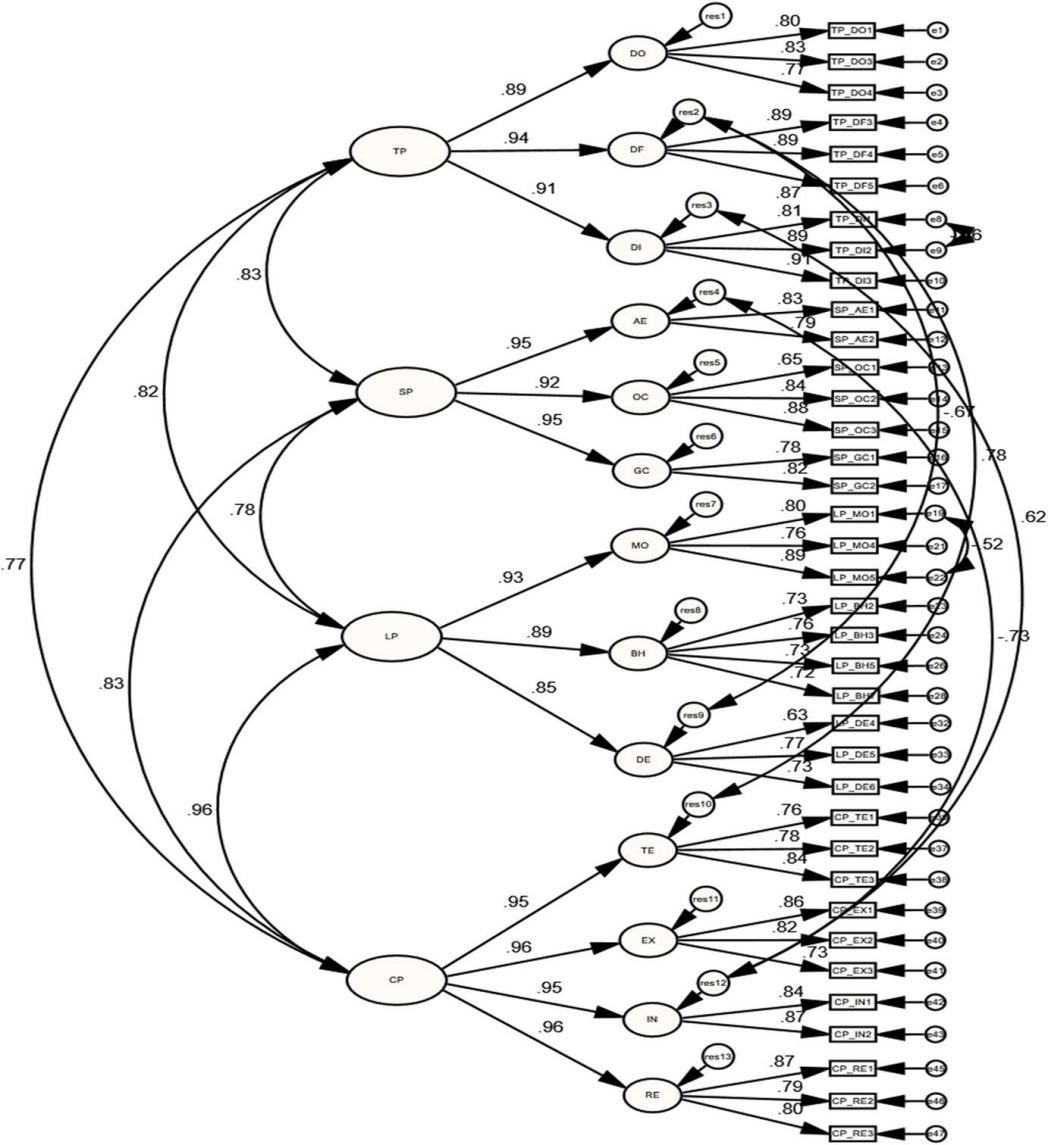
Standardized estimates of the final higher-order COI measurement model

As can be seen in Fig. 5, Tables 4 and 5, indicators of the final higher and lower orders of COI measurement models included high and significant factor loadings. Results also revealed strong correlation estimates between TP with SP ($r=0.829$, $p<0.05$), LP ($r=0.816$, $p<0.05$), and CP ($r=0.772$, $p<0.05$), as explained in Table 6. Furthermore, SP had strong correlation estimates with LP ($r=0.784$, $p<0.05$) and CP ($r=0.831$, $p<0.05$). Likewise, a strong correlation estimate was found between LP and CP ($r=0.958$, $p<0.01$).

**Table 4:** CFA, reliability, validity, and discriminant validity results of the higher-order COI measurement model

HOCs	LOCs	Number of items	Loadings	Bootstrapping CI 95%		p	CR	α	AVE	VIF
				Lower	Upper					
TP	DO	3	0.894**	0.835	0.945	0.005	0.94	0.94	0.84	2.50
	DF	3	0.941**	0.918	0.968	0.006				
	DI	3	0.910*	0.845	0.953	0.012				
SP	AE	2	0.951**	0.898	0.994	0.005	0.96	0.90	0.88	2.40
	OC	3	0.922**	0.860	0.979	0.008				
	GC	2	0.952**	0.900	0.989	0.009				
LP	MO	3	0.929*	0.879	0.955	0.023	0.92	0.91	0.79	2.04
	BH	4	0.892*	0.808	0.932	0.030				
	DE	3	0.846*	0.705	0.913	0.025				
CP	TE	3	0.953**	0.928	0.983	0.006	0.97	0.95	0.91	
	EX	3	0.963**	0.944	0.987	0.005				
	IN	2	0.949**	0.917	0.980	0.007				
	RE	3	0.958*	0.934	0.981	0.011				

$$N=203$$; *$$p<0.05$$; **$$p<0.01$$

**Table 5:** CFA, reliability, and validity results of the lower-order COI measurement model

LOCs	Item code	Items	Loadings	Mean	SD	Bootstrapping CI 95%		p	CR	α	AVE
						Lower	Upper				
DO									0.843	0.836	0.642
	TP_DO1	The instructor clearly communicated important course topics	0.803**	4.22	0.841	0.719	0.894	0.005			
	TP_DO3	The instructor provided clear instructions on how to participate in course learning activities	0.832*	4.24	0.818	0.767	0.885	0.020			
	TP_DO4	The instructor clearly communicated important due dates/time frames for learning activities	0.767*	4.21	0.975	0.674	0.851	0.012			
DF									0.914	0.912	0.781
	TP_DF3	The instructor helped to keep course participants engaged and participating in productive dialogue	0.892*	4.12	1.000	0.845	0.926	0.014			
	TP_DF4	The instructor helped keep the course participants on task in a way that helped me to learn	0.890*	4.27	0.923	0.821	0.927	0.016			
	TP_DF5	The instructor encouraged course participants to explore new concepts in this course	0.869*	4.27	0.959	0.787	0.918	0.013			
DI									0.906	0.903	0.764
	TPDI1	My instructor provided useful illustrations that helped make the course content more understandable to me	0.812**	4.20	0.919	0.722	0.879	0.009			
	TP_DI2	My instructor presented helpful examples that allowed me to better understand the content of the course	0.894*	4.30	0.903	0.836	0.937	0.012			
	TP_DI3	My instructor provided clarifying explanations or other feedback that allowed me to better understand the content of the course	0.913*	4.20	0.955	0.861	0.954	0.015			
AE									0.787	0.834	0.649
	SP_AE1	Getting to know other course participants gave me a sense of belonging in the course	0.826**	4.00	1.036	0.747	0.896	0.009			
	SP_AE2	I was able to form distinct impressions of some course participants	0.785*	4.00	0.972	0.675	0.852	0.025			
OC									0.837	0.815	0.634
	SP_OC1	I felt comfortable conversing through the online medium	0.654*	3.97	0.990	0.536	0.740	0.023			
	SP_OC2	I felt comfortable participating in the course discussions	0.841*	4.08	0.989	0.765	0.903	0.010			
	SP_OC3	I felt comfortable interacting with other course participants	0.876*	4.00	1.022	0.811	0.936	0.011			
GC									0.784	0.777	0.644
	SP_GC1	I felt comfortable disagreeing with other course participants while still maintaining a sense of trust	0.781*	3.81	1.084	0.655	0.854	0.019			
	SP_GC2	I felt that my point of view was acknowledged by other course participants	0.824*	4.01	0.992	0.706	0.893	0.026			
MO									0.858	0.824	0.670
	LP_MO1	I will be able to use what I learn in this course in my career	0.805**	4.12	0.985	0.705	0.872	0.007			
	LP_MO4	I am confident I can do an excellent job on the assignments and tests in this course	0.758*	4.14	1.000	0.671	0.824	0.021			
	LP_MO5	I can master the skills being taught in this class	0.887*	4.10	0.944	0.816	0.939	0.015			
BH									0.826	0.817	0.542
	LP_ BH2	I study in a place where I can concentrate on my course work	0.732**	4.18	0.878	0.626	0.832	0.009			
	LP_ BH3	I keep up with all the weekly readings and assignments for this course	0.765*	4.07	0.975	0.636	0.834	0.020			
	LP_ BH5	I set goals to help me manage my time for my online course	0.729**	4.05	1.004	0.642	0.815	0.009			
	LP_ BH7	I rely on comments from my instructor for feedback on how well I am doing in the course	0.719*	4.12	0.921	0.632	0.792	0.014			
DE									0.755	0.782	0.509
	LP_ DE4	I prefer courses that focus on discussions of personal answers based on evidence rather than just right and wrong answers	0.633*	4.03	0.917	0.452	0.741	0.023			
	LP_ DE5	I prefer courses where my final grade is based mostly on how well I can synthesize and practically apply various course topics in a real-world context that goes beyond what was directly taught in the course	0.769*	4.06	0.944	0.668	0.834	0.023			
	LP_ DE6	I prefer instructors who challenge me to develop my own opinions that are supported by evidence	0.731*	4.01	0.912	0.587	0.831	0.019			
TE									0.835	0.835	0.628
	CP_TE1	Problems posed increased my interest in course issues	0.758*	3.92	0.956	0.634	0.843	0.012			
	CP_TE2	Course activities piqued my curiosity	0.778*	3.98	0.949	0.673	0.851	0.013			
	CP_TE3	I felt motivated to explore content related questions	0.839*	4.05	0.929	0.732	0.878	0.046			
EX									0.848	0.835	0.651
	CP_EX1	I utilized a variety of information sources to explore problems posed in this course	0.858*	3.96	0.943	0.793	0.899	0.018			
	CP_EX2	Brainstorming and finding relevant information helped me resolve content related questions	0.822*	4.17	0.898	0.757	0.868	0.016			
	CP_EX3	Online discussions were valuable in helping me appreciate different perspectives	0.735*	4.00	0.954	0.641	0.810	0.010			
IN									0.844	0.850	0.730
	CP_IN1	Combining new information helped me answer questions raised in course activities	0.843*	4.15	0.839	0.784	0.891	0.014			
	CP_IN2	Learning activities helped me construct explanations/solutions	0.866*	4.09	0.958	0.795	0.904	0.032			
RE									0.861	0.858	0.675
	CP_RE1	I can describe ways to test and apply the knowledge created in this course	0.872*	4.06	0.882	0.820	0.919	0.013			
	CP_RE2	I have developed solutions to course problems that can be applied in practice	0.791**	3.94	0.944	0.718	0.859	0.009			
	CP_RE3	I can apply the knowledge created in this course to my work or other non-class related activities	0.799*	4.13	0.905	0.729	0.853	0.012			

$$N=203$$; $$SD=standard deviation$$; *$$p<0.05$$; **$$p<0.01$$

**Table 6:** Correlation estimates of the higher-order COI measurement model

Parameters	Estimate	Bootstrapping CI 95%		p
		Lower	Upper	
TP $\leftrightarrow$ SP	0.829*	0.756	0.892	0.020
TP $\leftrightarrow$ LP	0.816*	0.699	0.876	0.019
TP $\leftrightarrow$ CP	0.772*	0.695	0.848	0.015
SP $\leftrightarrow$ LP	0.784*	0.696	0.874	0.010
SP $\leftrightarrow$ CP	0.831*	0.737	0.910	0.019
LP $\leftrightarrow$ CP	0.958**	0.913	0.997	0.009

$$N=203$$; *$$p<0.05$$; **$$p<0.0$$

### The structural model of the higher-order COI framework

The structural model of the higher-order COI measurement model was also examined. As presented in Tables 4 and 5, results revealed that all composite reliability (CR), Cronbach’s alpha (α), and average variance extraction (AVE) values were above the threshold values of 0.70, 0.708, and 0.50, respectively, indicating that reliability and validity requirements had been established (Hair et al., 2013; Nunally & Bernstein, 1994). As presented in Table 4, all variance inflation factor (VIF) values TP ($VIF=2.50$), SP ($VIF=2.40$), and LP ($VIF=2.04$) were below the value of 10, revealing the absence of multicollinearity issues (Kutner et al., 2004). The discriminant validity between the subscales with their relevant main presences was not evaluated as the violation is expected (Sarstedt et al., 2019).

**Fig. 6:**
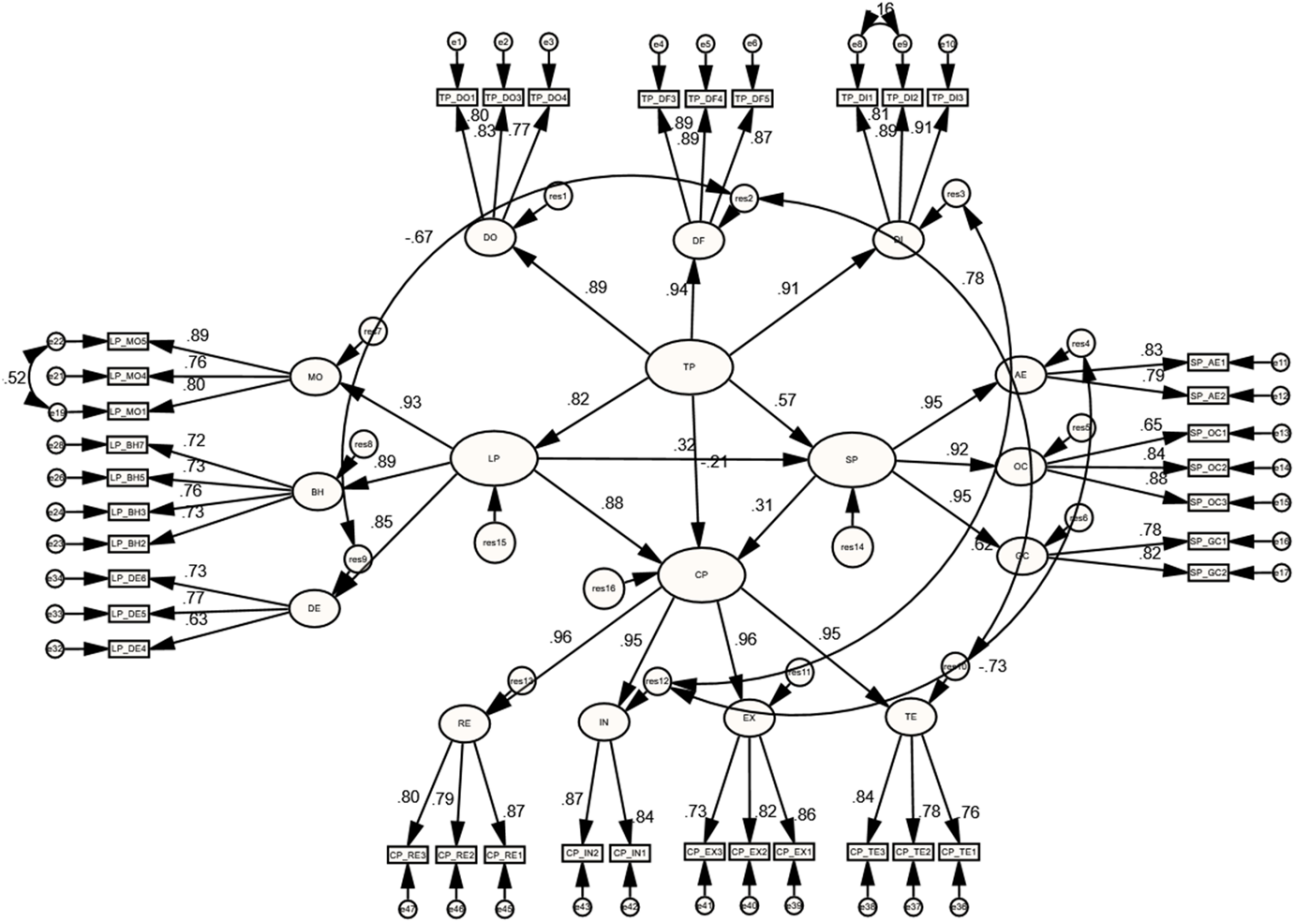
The structural model results of the higher-order COI

As shown in Fig. 6, Table 7, standardized coefficient (β) estimates demonstrated that CP was positively and significantly influenced by SP ($\upbeta =0.307$, $p<0.05$) and LP ($\upbeta =0.884$, $p<0.01$). Contrarily, TP had an insignificant influence on CP ($\upbeta =-0.205$, $p>0.05$). These three constructs (TP, SP, and LP) explained 77.9% of the variance in CP. Furthermore, SP was positively and significantly influenced by TP ($\upbeta =0.567$, $p<0.05$) and LP ($\upbeta =0.321$, $p<0.05$), explaining 58.4% of the variance in SP. Lastly, TP positively and significantly influenced LP ($\upbeta =0.816$, $p<0.05$), explaining 45.2% of the variance in LP.

**Table 7:** Standardized regression weights results of the higher-order COI structural model

Relationships	β	Bootstrapping CI 95%		P	${R}^{2}$
		Lower	Upper		
LP $\leftarrow$ TP	0.816*	0.699	0.876	0.019	0.452
SP $\leftarrow$ TP	0.567*	0.249	0.811	0.023	0.584
SP $\leftarrow$ LP	0.321*	0.074	0.626	0.015	
CP $\leftarrow$ TP	-0.205	-0.458	0.124	0.279	0.779
CP $\leftarrow$ SP	0.307*	0.089	0.615	0.013	
CP $\leftarrow$ LP	0.884**	0.650	1.135	0.008	

$$N=203$$; *$$p<0.05$$; **$$p<0.01$$

## Discussion

This study aimed to evaluate the validity and reliability of the extended COI framework using the CFA analysis. It firstly evaluated the measurement model of each lower-order COI presence. Results showed a mediocre model fit for each lower-order model after dropping three TP indicators, one SP indicator, nine LP indicators, and one CP indicator. High, positive, and significant correlation and standardized estimates of the relationships between the sub-dimensions of each lower-order model of COI presences were obtained. Thereafter, the higher-order measurement model of the extended COI framework was evaluated, including the four presences in a single model. It comprised ten TP indicators, eight SP indicators, ten SP indicators, and 11 CP indicators. Additional specifications were applied to the higher-order COI measurement model to meet the acceptable model fit criteria. The final model involved 37 indicators, with an acceptable model fit for the higher-order COI framework. In addition, the results of this study revealed positive and significant correlations and standardized estimates of the relationships between TP, SP, LP, and CP.

Furthermore, study findings revealed large effect sizes of the correlations between TP and SP ($d=2.965$), TP and LP ($d=2.823$), TP and CP ($d=2.429$), SP and LP ($d=2.526$), SP and CP ($d=2.988$), and even between LP and CP ($d=6.681$). These results are consistent with previous studies (Shea & Bidjerano, 2010, 2012; Traver et al., 2014; Wertz, 2014, 2022).The strong correlation between LP and CP implies that students’ self-regulatory skills have a significant impact on their CP which is the core of COI (Garrison et al., 1999). This finding is consistent with Wertz's (2022) finding which demonstrates the added value by adding LP to the COI framework. Overall, these results conclude that the four presences of the extended COI framework are dynamically and statistically correlated.

The validity of the extended higher-order COI measurement model was established. This was examined by the results of the higher-order measurement model fit, the AVE of main presences scales and their subscales, and the discriminant validity between the main COI presences. Findings indicated sufficient results, indicating the validity of the extended COI framework. Moreover, the reliability of the main presences and their subscales was evaluated by the composite reliability and Cronbach’s alpha estimates. Study findings revealed sufficient estimates, indicating that the reliability of the extended COI survey was established. These findings confirm that LP can be considered as the fourth presence of the COI framework, supporting the argument that the self-regulation process can be a part of the blended learning community of inquiry.

The structural path of the extended COI framework was also examined. Study findings demonstrated that TP positively influences LP and SP, indicating that increasing the instructor’s presence in blended learning increases students’ learning and social presence. In other words, efforts devoted by course instructors in designing the course, facilitating students’ learning, and providing them with the needed guidance and instructions contribute to rising students’ motivation, self-regulation behaviours, and intellectual development process. Additionally, this contributes to building a sense of community through increasing students’ expression of self to their fellow students, increasing their discussions, and the group cohesion as well. These findings confirm the COI perspective and are consistent with previous studies (Lee et al., 2021; Wertz, 2014; Zhang, 2020). Moreover, study findings confirm the idea that course instructors have important roles in learning environments which are mediated by computer technology (Eom & Ashill, 2016).

Surprisingly, study findings indicated that TP has an insignificant effect on CP. This result is contrary to a previous study which found that TP has a significant positive impact on CP (Lee et al., 2021; Zhang, 2020). One possible explanation for this finding is that students were obliged to deal with the circumstances of the COVID-19 pandemic by coping with blended learning despite the presence of course instructors. The blended learning approach was mostly applied in the Egyptian higher education institutions after the breakout of the pandemic, allowing students to complete their studies in a safe learning environment.

Results also demonstrated that LP significantly influences students’ SP and CP. This explains that increasing students’ motivation, self-regulation behaviours, and intellectual development contribute to increasing their expression to other students, encouraging their discussions, increasing the group cohesion, and rising their learning construction process. These findings are consistent with a previous study (Wertz, 2014). Finally, the current study indicated that SP significantly influences CP, indicating that increasing students’ CP contributes to increasing their CP. This is consistent with the COI concept and even with previous studies’ findings (Wertz, 2014; Zhang, 2020).

## Implications and future work

This study provides methodological and practical implications. Methodologically it adds a contribution to the literature by validating the multi-dimensional extended COI framework. This contribution allows researchers to employ structural path analysis and explore the inter-relationships between the extended COI presences. Practically, it provides meaningful investigations of the relationships between TP, SP, LP, and CP. Study findings can help practitioners in understanding the methods and strategies needed for building a community blended learning environment, specifically in a developing country such as Egypt where the blended learning experience remains in an initial stage (Adel, 2017). The findings suggest that help course instructors should devote more efforts to encouraging students’ social and learning presences. This can be achieved by designing community learning activities, encouraging students’ discussions, designing group activities, and allowing students to express their ideas and experiences (Dunlap & Lowenthal, 2018; Stephens & Roberts, 2017).

This study does, however, have several limitations. First, study findings cannot be generalised, and further work is required to be conducted and even with samples drawn from other geographic locations. Second, the sample size drawn in this study affects the model fit results, suggesting the need for further work with larger samples to obtain better results. Third, the COVID-19 pandemic has provided awareness of the role of students’ emotions which may affect their learning experience and attitudes. This could not be captured in the current study and further qualitative study can explore important insights regarding this matter.

## Data Availability

The datasets generated during and/or analysed during the current study are not publicly available due to privacy consideration but are available from the corresponding author on reasonable request.
